# Enhanced nonlinear magneto-optical rotation in cold atoms: A theoretical study

**DOI:** 10.1038/s41598-019-42710-z

**Published:** 2019-04-19

**Authors:** Mohsen Ghaderi Goran Abad, Mitra Valinezhad, Mohammad Mahmoudi

**Affiliations:** Department of Physics, University of Zanjan, University Blvd., 45371-38791 Zanjan, Iran

**Keywords:** Magneto-optics, Nonlinear optics

## Abstract

We theoretically investigate magneto-optical rotation (MOR) of a linearly polarized probe field in the four-level N-type cold atoms. By applying a static magnetic field and a weak coupling field, it is shown that the birefringence enhancement is induced in the system. Moreover, we show that the static magnetic field has a major role in switching the dichroism to enhanced birefringence in the system. We also obtain a large intensity for the output field with nearly perpendicular MOR angle by 88 degrees with subnatural width. It is demonstrated that Doppler broadening has a destructive effect on the MOR of the polarization direction of the probe field. The results of our study can be used for selecting narrow band of wavelengths and polarization converter for efficient switching of TM/TE polarization modes in optical communication, the depolarization backscattering lidar, polarization spectroscopy and precision measurements.

## Introduction

It is well known that the polarization plane of a linearly polarized field is rotated passing through an anisotropic medium. In fact, asymmetry between refractive indices of the circular components of the linear field is the cornerstone of the rotation of the polarization plane. When a medium is subjected to a magnetic field, Zeeman splitting of the magnetic degenerate sub-levels is the responsible for the asymmetry leading to the Faraday rotation^1,2^. Faraday and Voigt effects depend respectively on the longitudinal and transverse orientation of the external magnetic field with respect to the propagation direction of the light. In MOR, the optical fields interact with atoms in the presence of an externally static magnetic field. Agarwal *et al*. have reported the possibility of the control of MOR in atomic system using a coherent coupling field and the static magnetic^3^. The effect of temperature of the atomic system on MOR has been studied in multiple three-level electromagnetically induced transparency subsystems and the maximum rotation angle have been reported 45 degrees at T = 65 °C^4^. The coherent control of optical polarization has been studied in GaAs quantum well nanostructures^5^, metamaterials^6^, single- and multilayer graphene^7^, mesogenic organic molecules^8^, diamond nitrogen vacancy centers^9^ and cavity QED^10^. Moreover, several experimental studies have been reported in generation and control of the MOR^11–15^.

Numerous studies have reported that manipulating of the polarization of a field has a wide variety of application^16^ in magnetometry^17,18^ atomic clocks^19^, optical limitation^20,21^, optical atomic filters based on birefringence^22,23^ and dichroism^24^. One of the interesting applications of the MOR is magnetometry. The output of nonlinear MOR is a signal that depends on both light and static magnetic field. It allows us to accurate measurements of non-zero magnetic fields^25^. Petrosyan *et al*. proposed an experimentally feasible setup that can induce large MOR and therefore be used for ultrasensitive optical magnetometry^26^. The effect of the intensity and atomic density on the MOR have been reported in a three-level lambda-type system^27^. Another mechanism in removing degeneracy and making asymmetry is based on the AC-stark shift^28^. It was shown that a circularly polarized control light plays the role of a static magnetic field via a Zeeman-like ac Stark interaction and induces the optical Faraday rotation of the probe light^29^. Recently, the polarization rotation with the subnatural width, in the absence of the static magnetic field, has been reported at room temperature and the several 10^−3^ rad rotation has been measured in the hyperfine transition of the Rb vapor cell^30^. More recently, the effect of microwave field on the nonlinear optically induced Faraday rotation was presented and it was demonstrated that the optical activity due to the enhanced birefringence can be generated in a wide range of frequency^31^.

In this paper, MOR of a linearly polarized field is theoretically investigated passing through the four-level N-type cold atoms in the presence of a static magnetic field. It is shown that using a weak coupling field and a static magnetic field, a birefringence enhancement is induced in the system. We show that the linearly polarized probe field is transmitted through the medium by 0.94 of the intensity of the input field while its polarization direction is rotated up to 88 degrees due to the large birefringence induced in a narrow subnatural linewidth. We also find that the generation and control of the dichroism or birefringence in the system depend on the applied static magnetic field. Finally, the effect of the Doppler broadening is studied on the MOR and it is observed that increase of the Doppler width has a destructive role in MOR and reduces dramatically the intensity of the output field. Despite the decrease in MOR by increasing the temperature, it is reported a MOR angle by 17 degrees at room temperature. Our obtained results can be used for designing a monochromator as well as in the TM/TE polarization converters in optical communication, the depolarization backscattering lidar, polarization spectroscopy and precision measurements.

## Theoretical framework

**Figure 1:**
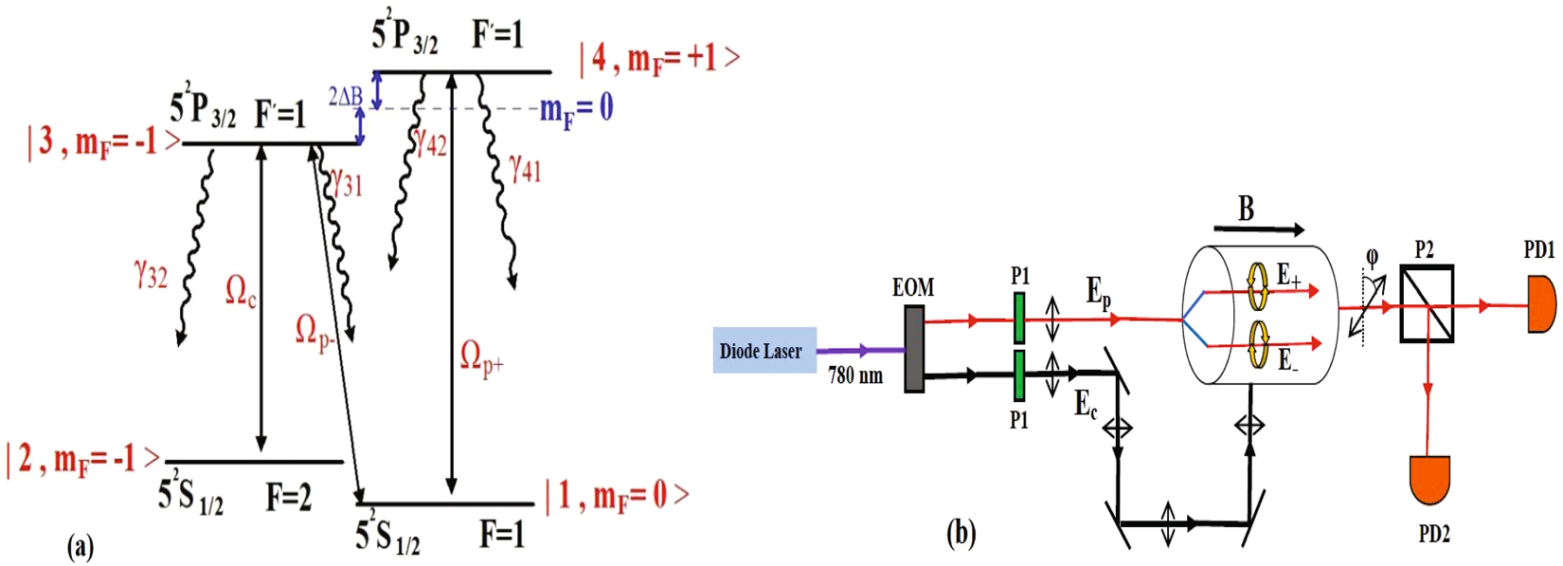
(**a**) Schematic diagram of a four-level N-type system. The system is driven by a weak coupling field with Rabi frequency ${{\rm{\Omega }}}_{c}$ and a right- and left- circularly polarized fields derived from a linearly polarized probe field with Rabi frequencies ${{\rm{\Omega }}}_{p+}$ and ${{\rm{\Omega }}}_{p-}$ respectively. (**b**) Schematic of experimental setup. EOM is a electro-optical modulator, P1 a polarizer, P2 a polarization analyzer and PD1 and PD2 are photodiodes.

The considered system is a four-level N-type system shown schematically in Fig. 1. This system can be generated in *D*_2_ lines, including the transitions ${5}^{2}{S}_{1/2}\leftrightarrow {5}^{2}{P}_{3/2}$ of ^87^Rb atoms in a vapor medium. Two states $|1\rangle =|{5}^{2}{S}_{1/2},$ $(F=1,{m}_{F}=0)\rangle$ and $|2\rangle =|{5}^{2}{S}_{1/2},(F=2,{m}_{F}=-\,1)\rangle$, separated by 6.83 GHz are set to be the ground states and two degenerate states $|3\rangle =|{5}^{2}{P}_{3/2},(F^{\prime} =1,{m}_{F}=-\,1)\rangle$ and $|4\rangle =|{5}^{2}{P}_{3/2},(F^{\prime} =1,{m}_{F}=1)\rangle$ are assumed as the excited states. Here, *F* and *F*′ are the quantum numbers of the total angular momentum and *m*_*F*_ denotes magnetic quantum number of the corresponding states. An externally static magnetic field $\overrightarrow{B}=B\,\hat{z}$ is applied to the medium that it removes the degeneracy of the excited levels $\mathrm{|3}\rangle$ and $\mathrm{|4}\rangle$ inducing a level splitting 2Δ*B*. On the Faraday geometry, a strong linearly polarized probe field $\overrightarrow{E}=\hat{x}{E}_{p}\exp [\,-\,i({\omega }_{p}t-{k}_{p}z)]+c.c$ is applied to the medium parallel to the applied magnetic field. Since a linearly polarized probe field is a linear combination of right- and left- circularly polarized fields, the right- (left-) circular component of the linear probe field couples the transition $|4\rangle \leftrightarrow |1\rangle$ ($|3\rangle \leftrightarrow |1\rangle$) with the Rabi frequency ${{\rm{\Omega }}}_{p+}=({\vec{\mu }}_{41}.{\hat{\epsilon }}_{+}){E}_{+}/\hslash$ (${{\rm{\Omega }}}_{p-}=({\vec{\mu }}_{31}.{\hat{\epsilon }}_{-}){E}_{-}/\hslash$). The excited state $\mathrm{|3}\rangle$ is coupled to the ground state $\mathrm{|2}\rangle$ with a linearly weak coupling field with the Rabi frequency ${{\rm{\Omega }}}_{c}=({\vec{\mu }}_{32}.{\hat{\epsilon }}_{c}){E}_{c}/\hslash$. Here, ${E}_{+}={E}_{-}={E}_{p}/\sqrt{2}$ and $|{\vec{\mu }}_{41}|=|{\vec{\mu }}_{31}|$. also, ${\varepsilon }_{i}(i=\pm ,c)$ are the unit vectors of the right- and left- rotating and weak coupling fields. Zeeman splitting of the levels $\mathrm{|3}\rangle$ and $\mathrm{|4}\rangle$ is given by $\hslash {{\rm{\Delta }}}_{B}={m}_{s}{g}_{s}{\mu }_{B}B$ where *μ*_*B*_ is Bohr magneton, *g*_*s*_ is Lande’ factor and ${m}_{s}=\pm \,1$ is magnetic quantum numbers of the corresponding sublevels of the excited states. A schematic of experimental setup is displayed in Fig. 1(b). A diode laser at 780 nm passes through a Electro-Optic Modulator (EOM) to generate the probe and coupling fields. Each of them propagates through a high-quality polarizer (P1) to have linear polarization. Then, the coupling field is set to pass through the medium perpendicular to the probe field. It is needed to say that perpendicular arrangement of the probe and coupling field is suitable for cold atoms^26^. Simultaneously, a static magnetic field is applied to the medium parallel to the probe field. The transmitted probe field is then passed through a polarization analyzer cube (P2). Finally, the transmitted probe fields with different polarizations are detected by PhotoDiodes PD1 and PD2 from both channels of P2. It allows simultaneous measurements of the polarization rotation angle and the transmitted fields intensities.

Under the dipole and the rotating wave approximation, the Hamiltonian describing system in the interaction picture is:

1 $$\begin{array}{rcl}{V}_{I} & = & -\hslash ({{\rm{\Omega }}}_{p+}^{\ast }{e}^{-i({{\rm{\Delta }}}_{p+}+{{\rm{\Delta }}}_{B})t}|1\rangle \langle 4|+{{\rm{\Omega }}}_{p-}^{\ast }{e}^{-i({{\rm{\Delta }}}_{p-}-{{\rm{\Delta }}}_{B})t}|1\rangle \langle 3|\\  &  & +\,{{\rm{\Omega }}}_{c}^{\ast }{e}^{-i({{\rm{\Delta }}}_{c}-{{\rm{\Delta }}}_{B})t}|2\rangle \langle 3|)+h.c.,\end{array}$$

where ${{\rm{\Delta }}}_{p+}={\omega }_{41}-{\omega }_{p+}$, ${{\rm{\Delta }}}_{p-}={\omega }_{31}-{\omega }_{p-}$ and ${{\rm{\Delta }}}_{c}={\omega }_{32}-{\omega }_{c}$ are the detunings of the applied fields and the related central transition frequencies. The density matrix equations of motion of the considered four-level *N*-type system can be written as

2 $$\begin{array}{ccc}{\dot{\rho }}_{11} & = & {\gamma }_{31}{\rho }_{33}+{\gamma }_{41}{\rho }_{44}+i{{\rm{\Omega }}}_{p-}^{\ast }{\rho }_{31}-i{{\rm{\Omega }}}_{p-}{\rho }_{13}+i{{\rm{\Omega }}}_{p+}^{\ast }{\rho }_{41}-i{{\rm{\Omega }}}_{p+}{\rho }_{14},\\ {\dot{\rho }}_{22} & = & {\gamma }_{32}{\rho }_{33}+{\gamma }_{42}{\rho }_{44}+i{{\rm{\Omega }}}_{c}^{\ast }{\rho }_{32}-i{{\rm{\Omega }}}_{c}{\rho }_{23},\\ {\dot{\rho }}_{44} & = & -({\gamma }_{41}+{\gamma }_{42}){\rho }_{44}+i{{\rm{\Omega }}}_{p+}{\rho }_{14}-i{{\rm{\Omega }}}_{p+}^{\ast }{\rho }_{41},\\ {\dot{\rho }}_{13} & = & -[({\gamma }_{31}+{\gamma }_{32})/2-i({{\rm{\Delta }}}_{p-}-{{\rm{\Delta }}}_{B})]{\rho }_{13}\\  &  & +\,i{{\rm{\Omega }}}_{p+}^{\ast }{\rho }_{43}-i{{\rm{\Omega }}}_{c}^{\ast }{\rho }_{12}+i{{\rm{\Omega }}}_{p-}^{\ast }({\rho }_{33}-{\rho }_{11}),\\ {\dot{\rho }}_{14} & = & -[({\gamma }_{41}+{\gamma }_{42})/2-i({{\rm{\Delta }}}_{p+}+{{\rm{\Delta }}}_{B})]{\rho }_{14}\\  &  & +\,i{{\rm{\Omega }}}_{p-}^{\ast }{\rho }_{34}+i{{\rm{\Omega }}}_{p+}^{\ast }({\rho }_{44}-{\rho }_{11}),\\ {\dot{\rho }}_{23} & = & -[({\gamma }_{31}+{\gamma }_{32})/2-i({{\rm{\Delta }}}_{c}-{{\rm{\Delta }}}_{B})]{\rho }_{23}\\  &  & -i{{\rm{\Omega }}}_{p-}^{\ast }{\rho }_{21}+i{{\rm{\Omega }}}_{c}^{\ast }({\rho }_{33}-{\rho }_{22}),\\ {\dot{\rho }}_{24} & = & -[({\gamma }_{41}+{\gamma }_{42})/2-i({{\rm{\Delta }}}_{p+}-{{\rm{\Delta }}}_{p-}+{{\rm{\Delta }}}_{c}+{{\rm{\Delta }}}_{B})]{\rho }_{24}\\  &  & +\,i{{\rm{\Omega }}}_{c}^{\ast }{\rho }_{34}-i{{\rm{\Omega }}}_{p+}^{\ast }{\rho }_{21},\\ {\dot{\rho }}_{34} & = & -[({\gamma }_{31}+{\gamma }_{32}+{\gamma }_{41}+{\gamma }_{42})/2-i({{\rm{\Delta }}}_{p+}-{{\rm{\Delta }}}_{p-}\\  &  & +\,2{{\rm{\Delta }}}_{B})]{\rho }_{34}+i{{\rm{\Omega }}}_{p-}{\rho }_{14}+i{{\rm{\Omega }}}_{c}{\rho }_{24}-i{{\rm{\Omega }}}_{p+}^{\ast }{\rho }_{31},\\ {\dot{\rho }}_{12} & = & i({{\rm{\Delta }}}_{p-}-{{\rm{\Delta }}}_{c}){\rho }_{12}+i{{\rm{\Omega }}}_{p+}^{\ast }{\rho }_{42}+i{{\rm{\Omega }}}_{p-}{\rho }_{32}-i{{\rm{\Omega }}}_{c}{\rho }_{13},\\ {\dot{\rho }}_{33} & = & -({\dot{\rho }}_{11}+{\dot{\rho }}_{22}+{\dot{\rho }}_{44}),\end{array}$$

where ${\gamma }_{4i}({\gamma }_{3i})\,(i=1,2)$ is the decay rate of the excited level $|4\rangle$ $(|3\rangle )$ to the ground state $|i\rangle$. The response of the system to the right- and left- circular components of the probe field is determined by the susceptibilities which are given by

3 $${\chi }_{\pm }=(\frac{\alpha }{4\pi {k}_{p}}){S}_{\pm }$$

Here, *S*_±_ are the normalized susceptibilities defined as:

4 $${S}_{+}=\frac{{\rho }_{41}{\gamma }_{41}}{{{\rm{\Omega }}}_{p+}},\,{S}_{-}=\frac{{\rho }_{31}{\gamma }_{31}}{{{\rm{\Omega }}}_{p-}},$$

where *αl* = *4πk*_*p*_*lμ*^2^*N/ℏγ* is the field absorption at resonance in which *l* and *k*_*p*_ are the length of the atomic medium and probe field wave number respectively. Moreover, *N* indicates electron number density in the medium and $\mu =|{\vec{\mu }}_{41}|=|{\vec{\mu }}_{31}|$. The transition coherence of the right- and left- circular components of the probe field can be driven from equation (2). It should be noted that the real and imaginary parts of *S*_±_ represent the dispersion and absorption of the right- and left- circular component of the probe field. As mentioned above, it is assumed that the polarization direction of the input probe field is in $\hat{x}$ direction. So, the intensity of the output probe field in $\hat{y}$ direction is measured for calculating the MOR angle of the polarization of the probe field. In experimental works, it is done by using a $\hat{y}$-polarized analyzer permitting only a polarized field in $\hat{y}$ direction. Intensity of the output probe field with (without) rotated polarization direction *T*_*y*_ (*T*_*x*_) is given by^3^:

5 $${T}_{y}=\frac{{|{({E}_{{p}_{(out)}})}_{y}|}^{2}}{{|{E}_{{p}_{(in)}}|}^{2}}=\frac{1}{4}|\exp [i\alpha l{S}_{+}/2]-\exp [i\alpha l{S}_{-}/2]{|}^{2}$$

6 $${T}_{x}=\frac{{|{({E}_{{p}_{(out)}})}_{x}|}^{2}}{{|{E}_{{p}_{(in)}}|}^{2}}=\frac{1}{4}|\exp [i\alpha l{S}_{+}/2]+\exp [i\alpha l{S}_{-}/2]{|}^{2}$$

as well as the MOR angle is specified by

7 $$\varphi ={ta}{{n}}^{-1}[\sqrt{{T}_{y}/{T}_{x}}]={ta}{{n}}^{-1}(\pi {k}_{p}l({\chi }_{+}-{\chi }_{-}))$$

Rotation of the polarization plane of the probe field passing through a medium can be resulted in either birefringence or dichroism induced in the system. Difference between the real (imaginary) part of the normalized susceptibilities *S*_±_ leads to difference between the dispersions (absorptions) of the right- and left- circular components of the linearly polarized probe field making the medium to show birefringence (dichroism) behavior. It is noteworthy that to obtain the lossless MOR, the birefringence is preferred with respect to the dichroism. In current study, we try to enhance the birefringence and reduce the dichroism in the medium. Here, we are going to determine the suitable conditions for birefringence enhancement in the system. An interesting case takes place when ${\rm{Re}}[{S}_{+}]\ne {\rm{Re}}[{S}_{-}]$ and ${\rm{Im}}[{S}_{+}]={\rm{Im}}[{S}_{-}]=\beta$ occur simultaneously at which the MOR happens merely due to the birefringence. In this case, equations (5 and 6) are rewritten as

8 $${T}_{y}=\frac{{e}^{-\alpha l\beta }}{4}|\exp [i\alpha l{\rm{Re}}[{S}_{+}]/2]-\exp [i\alpha l{\rm{Re}}[{S}_{-}]/2]{|}^{2},$$

9 $${T}_{x}=\frac{{e}^{-\alpha l\beta }}{4}|\exp [i\alpha l{\rm{Re}}[{S}_{+}]/2]+\exp [i\alpha l{\rm{Re}}[{S}_{-}]/2]{|}^{2}.$$

when *β* is positive and $\alpha l\beta \ll 1$, the intensity of the probe field is not attenuated passing through the medium. In this case, the probe field rotates only due to the birefringence induced in the medium. However, the situation is different for negative *β* in which the propagating probe field experiences a gain, leading to amplification of the rotated probe field.

## Results and Discussion

**Figure 2:**
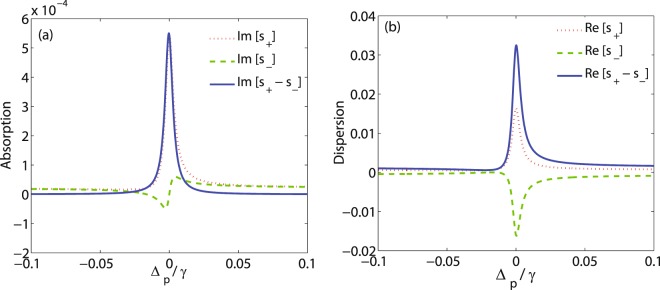
The imaginary (**a**) and real (**b**) parts of *S*_+_ (dotted) and *S*_−_ (dashed) and their difference (solid) versus detuning of the probe field Δ_*p*_. The other parameters are ${{\rm{\Omega }}}_{p}=0.5\gamma$, ${{\rm{\Omega }}}_{c}=0.1\gamma$, ${{\rm{\Delta }}}_{B}=30\gamma$, $\alpha l=190\gamma$ and ${{\rm{\Delta }}}_{c}=0$.

**Figure 3:**
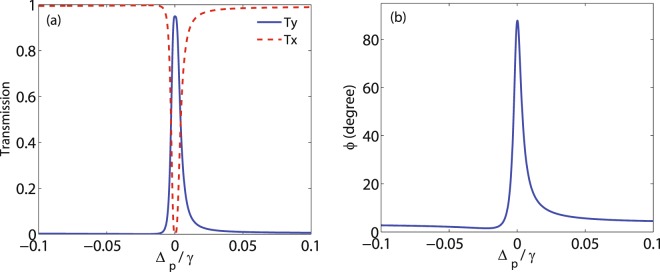
The intensity of the output field (**a**) in direction $\hat{y}$ (*T*_*y*_) (solid) and direction $\hat{x}$ (*T*_*x*_) (dashed) and MOR angle (**b**) versus detuning of the probe field Δ_*p*_. The other parameters are the same used in Fig. 2.

In this section, we study MOR of the polarization direction of a linearly polarized probe field crossing through a medium by numerically solving the equation (2) in the steady state. Throughout the results, the parameters used are scaled with *γ* which is taken as $\gamma =2\pi \times 6\,{\rm{MHz}}$ for *D*_2_ transition of ^87^Rb. Furthermore, it is supposed that ${{\rm{\Omega }}}_{p+}={{\rm{\Omega }}}_{p-}={{\rm{\Omega }}}_{p}$ and ${{\rm{\Delta }}}_{p+}={{\rm{\Delta }}}_{p-}={{\rm{\Delta }}}_{p}$. In Fig. 2(a and b), respectively, the imaginary and real parts of *S*_+_ (dotted), *S*_−_ (dashed) and their difference (solid) are plotted versus detuning of the probe field Δ_*p*_. The other parameters are ${{\rm{\Omega }}}_{p}=0.5\gamma$, ${{\rm{\Omega }}}_{c}=0.1\gamma$, ${{\rm{\Delta }}}_{B}=30\gamma$, $\alpha l=190\gamma$ and ${{\rm{\Delta }}}_{c}=0$. It is seen from Fig. 2(a) that when the probe field is tuned to resonance (${{\rm{\Delta }}}_{p}=0$), the absorption of the right- circular component is negligible $({\rm{Im}}[{S}_{+}]=0.0005)$, while the left- circular component is not absorbed. Meanwhile as can be seen in Fig. 2(b), the real parts of *S*_+_ and *S*_−_ are maximally different in magnitude and different in sign. Difference in dispersions of the right- and left- circular components of the probe field, accompanied by nearly zero absorption, implies a birefringence enhancement in the system at ${{\rm{\Delta }}}_{p}=0$. The intensity of the output field (a) in direction $\hat{y}$ (solid) and direction $\hat{x}$ (dashed) as well as the MOR angle (b) are displayed in Fig. 3 versus detuning of the probe field Δ_*p*_. It should be noted that *T*_*y*_ is the intensity of the output field with rotated polarization direction, while *T*_*x*_ is the intensity of the output field in input polarization direction. Figure 3(a) shows that *T*_*y*_ enhances around ${{\rm{\Delta }}}_{p}=0$ and reaches its peak in the resonance condition with a value of 0.94, while *T*_*x*_ attenuates extremely at ${{\rm{\Delta }}}_{p}=0$. It is known that measuring the intensity of the output field in direction $\hat{y}$ is the experimental method for measuring polarization rotation of the input field polarized in direction $\hat{x}$. The large intensity obtained in direction $\hat{y}$ for the output field (${T}_{y}=0.94$) indicates an enhancement in MOR. Moreover, as shown in Fig. 3(b), the MOR angle of the polarization direction of the probe field experiences a large rotation and its value reaches 88 degrees (nearly perpendicular) at ${{\rm{\Delta }}}_{p}=0$. Due to inducing the birefringence at ${{\rm{\Delta }}}_{p}=0$ mentioned in Fig. 2, the large obtained MOR of the polarization direction of the probe field is merely due to the birefringence induced in the system. Since ${\rm{Re}}[{S}_{+}-{S}_{-}]$ is positive in Fig. 2(b), equation (7) also represents that the direction of MOR of the polarization of the probe field is clockwise. It should be noted that the Zeeman splitting ${{\rm{\Delta }}}_{B}=30\gamma$ is corresponded to the static magnetic field $B\approx 64.5\,G$ which can be easily supplied in experimental works. A bird’s eye view of the Fig. 3(b) shows that the width of the MOR angle plot is narrower than natural linewidth of the probe field. This implies that the polarization of very narrow frequency linewidth can rotate to the direction $\hat{y}$. Then the suggested MOR model can be also used as a monochromator which transmits selectable narrow band of wavelengths of the probe field with resolving power $\frac{\omega }{{\rm{\Delta }}\omega }\approx {10}^{9}$.

**Figure 4:**
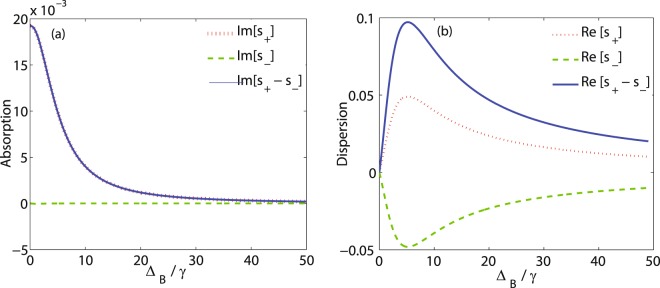
The imaginary (**a**) and real (**b**) parts of *S*_+_ (dotted) and *S*_−_ (dashed) and their difference (solid) versus Δ_*B*_. The other used parameters are ${{\rm{\Omega }}}_{p}=0.5\gamma$, ${{\rm{\Omega }}}_{c}=0.1\gamma$, $\alpha l=190\gamma$, ${{\rm{\Delta }}}_{p}=0$ and ${{\rm{\Delta }}}_{c}=0$.

In Fig. 4, the effect of the applied static magnetic field is studied on the MOR of the linearly polarized probe field. The imaginary (a) and real (b) parts of *S*_+_ (dotted), *S*_−_ (dashed) and their difference (solid) are plotted versus Δ_*B*_. The other parameters are considered as ${{\rm{\Omega }}}_{p}=0.5\gamma$, ${{\rm{\Omega }}}_{c}=0.1\gamma$, $\alpha l=190\gamma$, ${{\rm{\Delta }}}_{p}=0$ and ${{\rm{\Delta }}}_{c}=0$. It is shown that in the absence of the static magnetic field, the imaginary part of *S*_+_ and *S*_−_ is different. But the situation is different in the presence of the static magnetic field and their difference gradually decreases by increasing the static magnetic field. For the region of higher Zeeman splitting i.e., ${{\rm{\Delta }}}_{B} > 20\gamma$, absorptions of the right- and left- circular components of the probe field are negligible, so this region has a higher potential to investigate the birefringence enhancement in the system. Meanwhile, as shown in Fig. 4(b) when the static magnetic field is switched off, the dispersion of the probe field becomes zero. Thus, according to equation (7), birefringence cannot be induced in the system in the absence of the static magnetic field and dichroism is the dominant phenomenon. An investigation on Fig. 4(b) shows that the difference between two dispersions dramatically grows after switching on the static magnetic field. Thus, it can be said that the birefringence induced completely depends on the static magnetic field. Moreover, it is observed that the birefringence is the dominant phenomenon for higher values of Zeeman splitting, i.e. ${{\rm{\Delta }}}_{B} > 20\gamma$.

**Figure 5:**
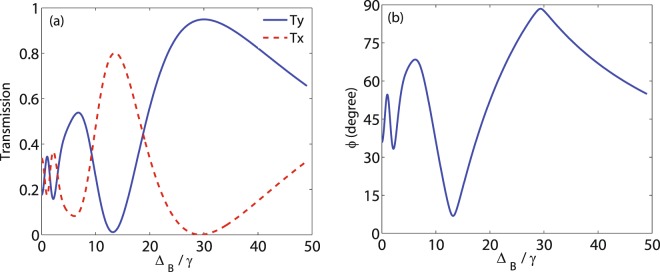
Intensity of the output field (**a**) in direction $\hat{y}$ (solid) and direction $\hat{x}$ (dashed) and MOR angle (**b**) versus Δ_*B*_. The other parameters are the same used in Fig. 4.

Let us now investigate the behavior of the output probe field for different values of the static magnetic field. Figure 5 shows the intensity of the output probe field (a) in directions $\hat{y}$ (solid) and $\hat{x}$ (dashed) as well as MOR angle (b) as a function of Δ_*B*_. It is seen from Fig. 5(a) that in the completely birefringent region (${{\rm{\Delta }}}_{B} > 20\gamma$), *T*_*y*_ enhances while *T*_*x*_ attenuates, leading to an enhancement in MOR angle as shown in Fig. 5(b). As a result, static magnetic field introduces a region in which MOR happens solely due to birefringence induced in the system. Note that for Zeeman splitting about ${{\rm{\Delta }}}_{B}=30\gamma$, a large intensity of the *T*_*y*_ (${T}_{y}=0.94$) as well as a large MOR angle ($\varphi \,=\,88$ degrees) are obtained for the polarization direction.

**Figure 6:**
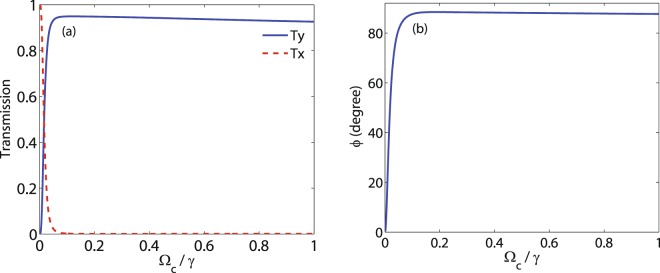
*T*_*y*_ (solid), *T*_*x*_ (dashed) (**a**) and MOR angle (**b**) versus ${{\rm{\Omega }}}_{c}$. The parameters used are ${{\rm{\Omega }}}_{p}=0.5\gamma$, ${{\rm{\Delta }}}_{B}=30\gamma$, $\alpha l=190\gamma$, ${{\rm{\Delta }}}_{p}=0$ and ${{\rm{\Delta }}}_{c}=0$.

In Fig. 6, the effect of the weak coupling field ${{\rm{\Omega }}}_{c}$ on the intensity of the output probe field (a) in direction $\hat{y}$ (solid) and direction $\hat{x}$ (dashed) and MOR angle (b) is investigated at ${{\rm{\Delta }}}_{p}=0$. The other used parameters are ${{\rm{\Omega }}}_{p}=0.5\gamma$, $\alpha l=190\gamma$, ${{\rm{\Delta }}}_{B}=30\gamma$ and ${{\rm{\Delta }}}_{c}=0$. It is shown in Fig. 6(a) that for small values of ${{\rm{\Omega }}}_{c}$, the intensity of the output field in direction $\hat{y}$ increases sharply and reaches a nearly maximum value. Simultaneously, *T*_*x*_ decreases to negligible value by increasing ${{\rm{\Omega }}}_{c}$. Figure 6(b) displays the behavior of the MOR angle versus ${{\rm{\Omega }}}_{c}$. It is expected that the similar behavior can be seen in the MOR angle. Note that the maximum MOR angle is not sensitive to the intensity fluctuations of the weak coupling field.

**Figure 7:**
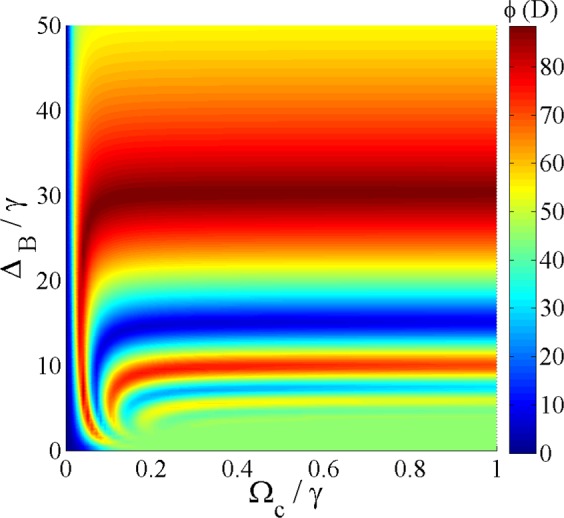
MOR angle versus ${{\rm{\Omega }}}_{c}$ and ${{\rm{\Delta }}}_{B}$. The parameters used are ${{\rm{\Omega }}}_{p}=0.5\gamma$, $\alpha l=190\gamma$, ${{\rm{\Delta }}}_{p}=0$ and ${{\rm{\Delta }}}_{c}=0$.

The effect of the different parameters on the MOR angle are simultaneously shown in Fig. 7. Figure 7 displays the density plot of the MOR angle as functions of ${{\rm{\Omega }}}_{c}$ and ${{\rm{\Delta }}}_{B}$. The parameters used are ${{\rm{\Omega }}}_{p}=0.5\gamma$, $\alpha l=190\gamma$, ${{\rm{\Delta }}}_{p}=0$ and ${{\rm{\Delta }}}_{c}=0$. An investigation on Fig. 7 shows that either the static magnetic field or weak coupling field can control the MOR angle. Thus, the suitable set of parameters can be determined for obtaining the different values of the MOR angle.

**Figure 8:**
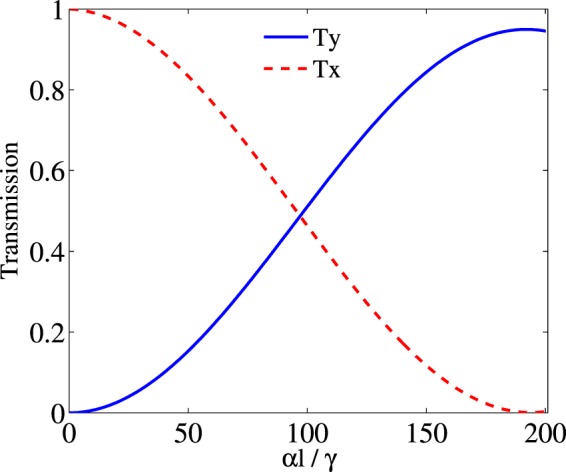
Evolutions of *T*_*x*_ (dashed) and *T*_*y*_ (solid) considered as TM and TE modes respectively versus *αl*. The other used parameters are ${{\rm{\Omega }}}_{p}=0.5\gamma$, ${{\rm{\Omega }}}_{c}=0.1\gamma$, ${{\rm{\Delta }}}_{p}=0$ and ${{\rm{\Delta }}}_{c}=0$.

The effect of the length of the atomic medium is another parameter which plays a major role in switching the intensity of the output probe field in direction $\hat{x}$ to direction $\hat{y}$. The evolutions of *T*_*y*_ and *T*_*x*_ are plotted versus *αl* in Fig. 8. The used parameters are ${{\rm{\Omega }}}_{p}=0.5\gamma$, ${{\rm{\Omega }}}_{c}=0.1\gamma$, ${{\rm{\Delta }}}_{B}=30\gamma$, ${{\rm{\Delta }}}_{p}=0$ and ${{\rm{\Delta }}}_{c}=0$. As can be seen in Fig. 8, by increasing the length of the medium, the intensity of the input probe field in direction $\hat{x}$ decreases and simultaneously the intensity of the output field increases in direction $\hat{y}$. Ultimately, it can be seen that for the higher values of *αl* i.e. $\alpha l=200\gamma$, the input probe field in $\hat{x}$ polarization is completely switched to $\hat{y}$ direction. So, our results can be used as a polarization converter for switching TM/TE modes.

Here, we are going to introduce an analytical expression to the physical interpretations of our numerical results. The normalized susceptibilities (*S*_±_), at resonance and weak coupling approximation, are given by

10 $${S}_{+}=\frac{\gamma (i\gamma +{{\rm{\Delta }}}_{B}){{\rm{\Omega }}}_{c}^{2}{{\rm{\Omega }}}_{p}(4{{\rm{\Omega }}}_{p}^{2}({{\rm{\Omega }}}_{c}^{2}+{{\rm{\Omega }}}_{p}^{2})+{{\rm{\Delta }}}_{B}{{\rm{\Delta }}}_{p}(2({{\rm{\Omega }}}_{c}^{2}+{{\rm{\Omega }}}_{p}^{2})+{{\rm{\Delta }}}_{B}{{\rm{\Delta }}}_{p}))}{{{\rm{\Omega }}}_{p}{{\rm{\Delta }}}_{B}^{2}(6{{\rm{\Omega }}}_{c}^{4}{{\rm{\Omega }}}_{p}^{2}+8{{\rm{\Omega }}}_{c}^{2}{{\rm{\Omega }}}_{p}^{4}+{{\rm{\Delta }}}_{B}({{\rm{\Omega }}}_{c}^{2}+2{{\rm{\Omega }}}_{p}^{2}){{\rm{\Delta }}}_{p}(2{{\rm{\Omega }}}_{c}^{2}+{{\rm{\Delta }}}_{B}{{\rm{\Delta }}}_{p}))},$$

11 $${S}_{-}=\frac{-{{\rm{\Omega }}}_{c}^{2}{{\rm{\Omega }}}_{p}(2{{\rm{\Omega }}}_{p}^{2}+{{\rm{\Delta }}}_{B}{{\rm{\Delta }}}_{p})\,({{\rm{\Omega }}}_{c}^{2}+2{{\rm{\Omega }}}_{p}^{2}+(\,-\,i\gamma +{{\rm{\Delta }}}_{B}){{\rm{\Delta }}}_{p})}{{{\rm{\Omega }}}_{p}{{\rm{\Delta }}}_{B}(6{{\rm{\Omega }}}_{c}^{4}{{\rm{\Omega }}}_{p}^{2}+8{{\rm{\Omega }}}_{c}^{2}{{\rm{\Omega }}}_{p}^{4}+{{\rm{\Delta }}}_{B}({{\rm{\Omega }}}_{c}^{2}+2{{\rm{\Omega }}}_{p}^{2}){{\rm{\Delta }}}_{p}(2{{\rm{\Omega }}}_{c}^{2}+{{\rm{\Delta }}}_{B}{{\rm{\Delta }}}_{p}))}.$$

Equations (4 and 5) predict that the maximum MOR angles occur at

12 $${{\rm{\Delta }}}_{B}=\frac{\alpha l({{\rm{\Omega }}}_{c}^{6}+2{{\rm{\Omega }}}_{c}^{2}{{\rm{\Omega }}}_{p}^{2}A)\sqrt{-{(4An\pi {{\rm{\Omega }}}_{p}^{3})}^{2}+\alpha {l}^{2}{{\rm{\Omega }}}_{c}^{2}{({{\rm{\Omega }}}_{c}^{4}+2{{\rm{\Omega }}}_{p}^{2}A)}^{2}}}{4An\pi {{\rm{\Omega }}}_{p}{{\rm{\Omega }}}_{c}^{2}},$$

where $A=3{{\rm{\Omega }}}_{c}^{2}+4{{\rm{\Omega }}}_{p}^{2}$ and $n=1,2,3,\ldots$. In addition, the imaginary part of the *S*_+_ and *S*_−_, corresponding to the absorptions of the right- and left- circular components of the probe field, in the presence of the static magnetic field and at ${{\rm{\Delta }}}_{p}=0$ yields,

13 $${\rm{Im}}[{S}_{+}]=\frac{2{\gamma }^{2}({{\rm{\Omega }}}_{c}^{2}+{{\rm{\Omega }}}_{p}^{2})}{{{\rm{\Delta }}}_{B}^{2}(3{{\rm{\Omega }}}_{c}^{2}+4{{\rm{\Omega }}}_{p}^{2})},\,{\rm{Im}}[{S}_{-}]=0.$$

The above equation represents that the absorption of the right circular component is reduced by increasing the Zeeman splitting and left- circular component is not absorbed (see Fig. 3(a)). It corroborates that the presented analytical results are in good agreement with the obtained numerical results in Fig. 3 and Fig. 4.

Finally, we are interested in taking into account the effect of Doppler broadening on the MOR in the system. Through the numerical calculations, the effect of the Doppler broadening was ignored. In a sample, atoms may move with velocity *v*. Velocities of the atoms can influence the effective frequencies of the probe and coupling fields as ${\omega }_{p}-{k}_{p}.v$ and ${\omega }_{c}-{k}_{c}.v$. Here, *k*_*p*,*c*_ = +(−)*v*/*c* denotes propagation vector of the probe and weak coupling field with the same (opposite) direction of the atom motion. Throughout the results, we suppose that ${k}_{p}={k}_{c}=k$ as well as the probe and coupling field co-propagate in the same direction of the atom motion. Also, the detunings of the fields are replaced by ${{\rm{\Delta }}}_{p+}-k$.*v*, ${{\rm{\Delta }}}_{p-}-k$.*v* and ${{\rm{\Delta }}}_{c}-k$.*v* in equation (2). It is clear that the transition coherences ${\rho }_{41}$ and ${\rho }_{31}$ for the circular components of the probe field are dependent on velocity. Then normalized susceptibilities *S*_+_ and *S*_−_ are averaged over the Maxwell-Boltzmann distribution for velocities and are given by

14 $${S}_{+}={\int }_{-\infty }^{+\infty }\,\frac{{\rho }_{41}(kv)\gamma }{{{\rm{\Omega }}}_{p+}}f(kv)d(kv),$$

15 $${S}_{-}={\int }_{-\infty }^{+\infty }\,\frac{{\rho }_{31}(kv)\gamma }{{{\rm{\Omega }}}_{p-}}f(kv)d(kv),$$

where $f(kv)=\tfrac{1}{\sqrt{2\pi {D}^{2}}}{\exp }[\,-\,\tfrac{{v}^{2}}{{D}^{2}}]$ and $kD=\sqrt{\tfrac{2{k}_{B}T{\omega }_{p}^{2}}{m{c}^{2}}}$ is Doppler width of distribution. Here, *m* is mass of moving atom, *T* is temperature of the cell and *k*_*B*_ is Boltzmann constant.

**Figure 9:**
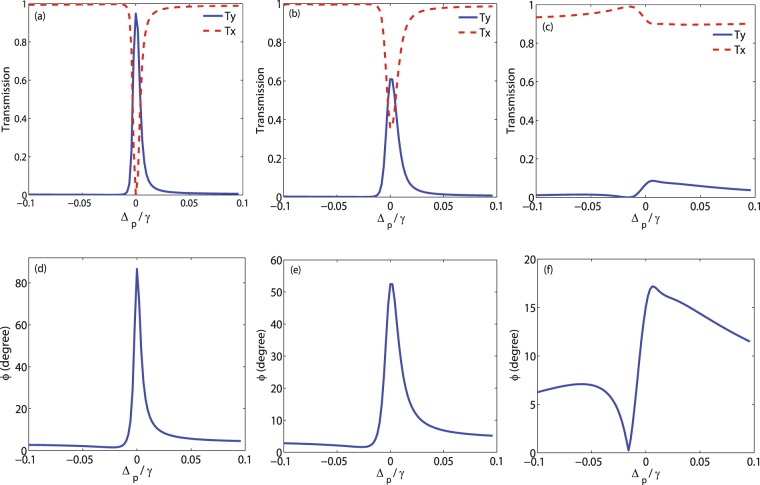
Intensity of the output field (upper row) in direction $\hat{y}$ (solid) and direction $\hat{x}$ (dashed), and MOR angle (lower row) variation versus detuning of the probe field for $kD=1\gamma$ (**a**,**d**), $kD=10\gamma$ (**b**,**e**) and $kD=50\gamma$ (**c**,**f**). The other parameters are the same used in Fig. 2.

In Fig. 9, the intensity of the output field in $\hat{y}$ (solid) and $\hat{x}$ (dashed) direction (upper row) and MOR angle of the polarization direction of the probe field (lower row) are plotted versus detuning of the probe field for different values of Doppler width $kD=\gamma$ (a,d), $kD=10\gamma$ (b,e) and $kD=50\gamma$ (c,f). Here, the Doppler width $kD=50\gamma$ corresponds to ^87^Rb vapor at room temperature. The other parameters are the same taken in Fig. 2. Our numerical results show that MOR is affected dramatically by Doppler broadening. It is observed from the upper row of the Fig. 9 that by increasing the Doppler width, *T*_*y*_ is extremely suppressed while *T*_*x*_ is enhanced at ${{\rm{\Delta }}}_{p}=0$.

**Figure 10:**
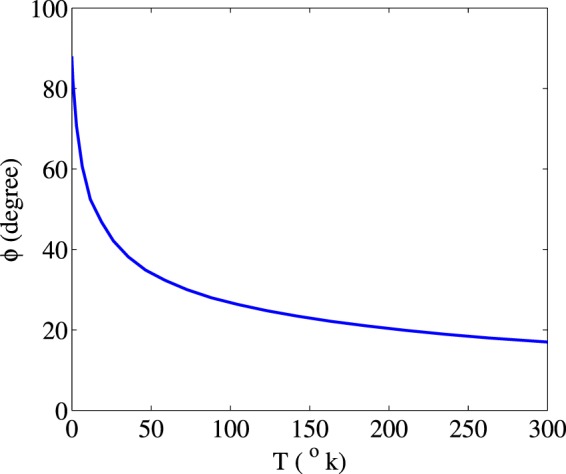
Behavior of MOR angle versus the temperature of the medium. The other taken parameters are ${{\rm{\Omega }}}_{p}=0.5\gamma$, ${{\rm{\Omega }}}_{c}=0.1\gamma$, ${{\rm{\Delta }}}_{B}=30$, $\alpha l=190\gamma$ and ${{\rm{\Delta }}}_{c}=0$.

The lower row of the Fig. 9 shows that the MOR angle of the polarization direction of the probe field drops strongly by increasing the Doppler width. Thereby, Doppler broadening has a destructive effect on the MOR of the polarization direction of the probe field. Moreover, it can be resulted that the enhancement in MOR is established only in cold atoms. The MOR angle behavior versus the temperature of the medium from the cold atoms to the room temperature is shown in Fig. 10. The used parameters are ${{\rm{\Omega }}}_{p}=0.5\gamma$, ${{\rm{\Omega }}}_{c}=0.1\gamma$, ${{\rm{\Delta }}}_{B}=30\gamma$ and ${{\rm{\Delta }}}_{c}=0$. It is seen that when atoms are cold, the polarization direction of the probe field experiences the maximum rotation. By increasing the temperature, MOR angle decreases to 17 degrees at room temperature.

## Conclusion

In summary, we have theoretically investigated the MOR of a linearly polarized probe field passing through the four-level *N*-type cold atoms. It was shown that in the presence of a static magnetic field and using a weak coupling field, a birefringence enhancement is induced in the system. We have illustrated that by applying the static magnetic field the dichroism switches to the enhanced birefringence in the system. Then, a higher intensity of the output field by 0.94 with roughly perpendicular rotated polarization direction by 88 degrees has been obtained with subnatural width. It was shown that the Doppler broadening has a destructive effect on the MOR so that MOR angle is reduced to 17 degrees at room temperature. The results can be used for designing a monochromator to select a wavelength with subnatural band width. Moreover, it can be used as a polarization converter for efficient switching of TM/TE polarization modes in optical communications.
